# Systematic review of machine learning in PTSD studies for automated diagnosis evaluation

**DOI:** 10.1038/s44184-023-00035-w

**Published:** 2023-09-27

**Authors:** Yuqi Wu, Kaining Mao, Liz Dennett, Yanbo Zhang, Jie Chen

**Affiliations:** 1 Electrical & Computer Engineering Department, Faculty of Engineering, University of Alberta, Edmonton, AB Canada; 2 Scott Health Sciences Library, University of Alberta, Edmonton, AB Canada; 3 Department of Psychiatry, Faculty of Medicine and Dentistry, University of Alberta, Edmonton, AB Canada

**Keywords:** Machine learning, Stress and resilience

## Abstract

Post-traumatic stress disorder (PTSD) is frequently underdiagnosed due to its clinical and biological heterogeneity. Worldwide, many people face barriers to accessing accurate and timely diagnoses. Machine learning (ML) techniques have been utilized for early assessments and outcome prediction to address these challenges. This paper aims to conduct a systematic review to investigate if ML is a promising approach for PTSD diagnosis. In this review, statistical methods were employed to synthesize the outcomes of the included research and provide guidance on critical considerations for ML task implementation. These included (a) selection of the most appropriate ML model for the available dataset, (b) identification of optimal ML features based on the chosen diagnostic method, (c) determination of appropriate sample size based on the distribution of the data, and (d) implementation of suitable validation tools to assess the performance of the selected ML models. We screened 3186 studies and included 41 articles based on eligibility criteria in the final synthesis. Here we report that the analysis of the included studies highlights the potential of artificial intelligence (AI) in PTSD diagnosis. However, implementing AI-based diagnostic systems in real clinical settings requires addressing several limitations, including appropriate regulation, ethical considerations, and protection of patient privacy.

## Introduction

Post-traumatic stress disorder (PTSD) is a debilitating condition that may arise after experiencing or witnessing a traumatic event^1^. Symptoms include recurrent memories, negative mood changes, altered arousal and reactivity, and avoidance of triggers, all of which can lead to significant mental and physical health problems, long-term disability, and socioeconomic burden^2^. Early diagnosis and intervention are essential for optimizing clinical outcomes and reducing direct and indirect costs associated with PTSD^3^. However, timely diagnosis is challenging due to the complex clinical manifestations and outdated diagnostic approaches^4^. Current diagnostic criteria rely on subjective assessment, while objective tests are currently unavailable due to the high cost and reliability concerns^1,5^. Moreover, the symptoms of PTSD may overlap with other disorders, making it difficult to establish a causal link. Therefore, reliable, sensitive, and easy-to-access approaches are needed to improve PTSD diagnosis.

The recent advent of artificial intelligence (AI) has provided a promising avenue to surmount current challenges, including improving the prediction and diagnosis of diseases such as PTSD. Machine learning (ML), a subset of AI, emphasizes the cultivation of algorithms and statistical models that enable computers to learn from data rather than operate under rigidly explicit instructions^6^. ML algorithms gain their proficiency by training on extensive datasets. Subsequently, they make predictions or decisions stemming from this training. The overarching aim of ML is to devise models capable of enhancing their precision over time and adeptly handling unseen data. ML encompasses multiple learning types, including supervised, unsupervised, semi-supervised, and reinforcement learning^7^. In supervised learning, algorithms are trained on datasets with known outputs. The algorithms are furnished with input data and the correct output to master a generalized correlation between the two. This enables the algorithm to predict unseen data via classification or regression. Established supervised learning methodologies, such as logistic regression (LR), decision trees (DT), and support vector machines (SVM), employ various statistical models to understand the input-output correlations and predict outcomes. To mitigate overfitting and augment accuracy, ensemble ML methods like random forest (RF) and gradient boosting (GB) have been introduced. Ensemble learning signifies an advanced strategy where a group of models is trained on a common problem. By integrating their results, both the performance and predictive capacity are considerably heightened, surpassing the capabilities of a single model^8–12^. Unsupervised learning, conversely, uncovers hidden patterns within unlabeled data, commonly used for clustering, dimensionality reduction, and feature extraction^13^. Analyses of more extensive, complex, and unstructured data, such as images or textual features, necessitate deep learning (DL) models.

**Fig. 1:**
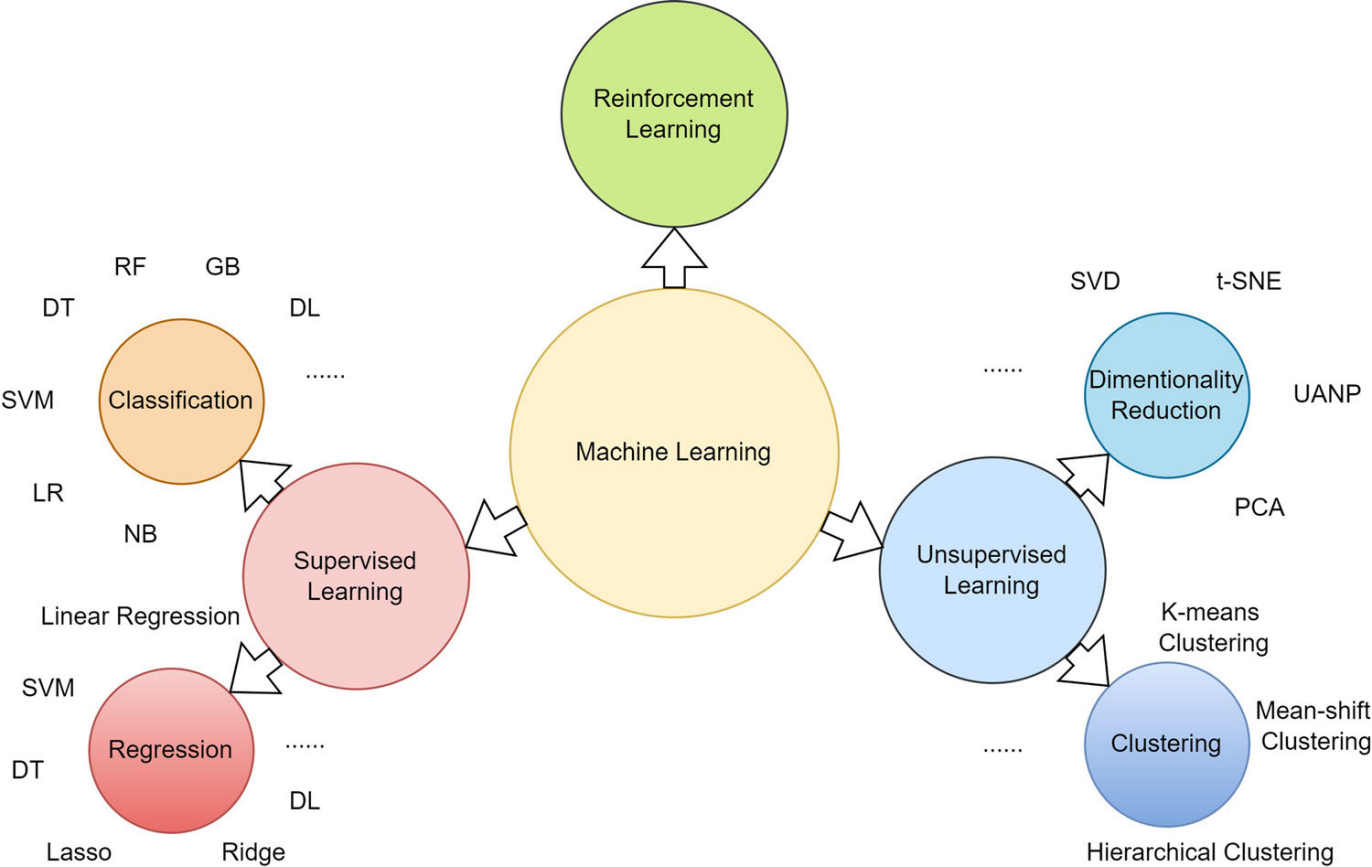
Machine learning model types. This figure introduced typical types of machine learning, including supervised learning, unsupervised learning, and reinforcement learning. In addition, most used models were listed based on specific tasks. For reinforcement learning, since it was not used in any included study, we did not explicitly introduce common models. Abbreviations: NB Naïve Bayes classifier, LR Logistic Regression classifier, SVM Support Vector Machine, DT Decision Tree classifier, RF Random Forest classifier, GB Gradient Boosting classifier, DL Deep Learning models, SVD Singular Value Decomposition, t-SNE t-Distributed Stochastic Neighbor Embedding, UANP Uniform Manifold Approximation and Projection, PCA Principal Component Analysis.

DL, an extended version of artificial neural networks (ANN), consists of multiple artificial neuron layers capable of learning more abstract data representations. DL algorithms, including convolutional neural networks (CNNs), recurrent neural networks (RNNs), and their variations, are frequently utilized in the medical domain^14–16^. Newly introduced architectures like transformers, designed for natural language processing (NLP) tasks, use a self-attention mechanism and encoder-decoder structure to scrutinize long-term dependencies in temporal samples, such as text or audio clips^17^. These features have been extensively adopted in the studies selected for this review and have yielded exceptional results in automated PTSD diagnosis. A brief introduction of various typical ML types and models is shown in the figure below (Fig.1*Machine learning model types)*.

Previous reviews have been conducted by various groups within this domain. Their scope predominantly encapsulated a summarization of existing studies, with a particular emphasis on the psychiatric aspects while missing comprehensive guideline to perform automated PTSD diagnosis task, which remains a critical need for a systematic examination of the computational science perspective on AI assisted PTSD^18,19^.

This paper presents a systematic review of 41 recent publications that apply AI methodologies for PTSD prediction and diagnosis, comparing the selection of models, datasets, and validation techniques used. Our systematic review aims to provide an exhaustive guide for researchers working in the AI-PTSD domain, thereby enhancing their understanding of the computational underpinnings and potential directions for future work. This guide provides strategic insights for the selection of models, the identification and selection of features, the choice and compilation of datasets, and the selection of evaluation metrics. These insights are not solely derived from evaluating the selected studies, rather they are also informed by the author’s background knowledge and prior experiences in automated mental disorder detection within other mental health domains. We have aimed to consolidate the knowledge base in this evolving field, facilitating more informed decision-making and contributing to advancements in AI-based PTSD prediction and diagnosis.

## Methods

### PRISMA guideline

This systematic review followed the Preferred Reporting Items for Systematic Reviews and Meta-Analyses (PRISMA) guidelines^20^. The PRISMA guidelines are designed to enhance transparency and completeness in reporting and to facilitate the critical appraisal and interpretation of systematic reviews and meta-analyses. Our review strategically incorporated items delineated in the PRISMA checklist, and these were subsequently adapted in accordance with the available data and the primary objectives of our review.

### Search strategy and literature selection

The search for articles for this review included databases such as MEDLINE, Embase, PsycINFO, Scopus, IEEE Xplore, and Compendex, covering the period between 1946 and 2022. The search was last conducted on October 18, 2022, using a query developed by LD, which included keywords and related terms for ‘PTSD,’ ‘machine learning,’ and ‘diagnosis.’ Various synonyms for ‘PTSD’ such as ‘PTSI,’ ‘PTSS,’ ‘post-traumatic,’ or ‘stress disorder’ were considered. In addition, ‘machine learning’ synonyms such as ‘artificial intelligence,’ ‘deep learning,’ ‘computer-assisted,’ ‘image classification,’ ‘computer vision,’ or ‘natural language processing’ were included. Papers with the keyword ‘prediction’ were considered synonymous with ‘diagnosis.’ The entire search filter can be found in the Supplementary Methods Query. Two rounds of study selection, namely title/abstract screening and full-text screening, were performed by two researchers, YW and KM, who independently evaluated each article based on the inclusion and exclusion criteria outlined in Table 1. In case of any conflicts, YW made the final decision.

**Table 1:** Eligibility criteria.

IC #	Inclusion Criteria (the following articles should be included):
IC1:	The article utilizes a machine learning model to conduct automatic diagnosis.
IC2:	The article pertains to PTSD diagnosis.
IC3:	The article explicitly mentions the evaluation metrics used to report the model’s performance.
IC4:	The article is authored in English.
EC #	Exclusion Criteria (the following articles should be excluded):
EC1:	Articles duplicated across various databases.
EC2:	Articles not related to the diagnosis or prediction of PTSD.
EC3:	Articles that do not report their evaluation methods and metrics.
EC4:	Articles focused on the prediction of future outcomes, risk factors of acquiring PTSD, or the trajectory of symptoms, rather than PTSD diagnosis.
EC5:	Articles primarily concerned with the feature selection process.
EC6:	Case studies.
EC7:	Articles not subjected to peer-review (non-journal articles).

### Data collection process

The selected studies were included for data extraction after passing the abstract/title and full-text screening. A pilot test was conducted on ten randomly chosen studies to validate the data extraction approach. YW extracted data from the selected studies, and KM checked the extracted data. Disagreements were resolved through discussion, and YW made the final decision. General information, such as first author, year of publication, title, technical information, and answers to the questions listed in Table 2, were extracted.

**Table 2:** Extraction questions.

EQ #	Extraction questions
EQ1:	What PTSD diagnostic method was used?
EQ2:	What was the data source? What was the sample distribution?
EQ3:	What was the best machine learning model reported?
EQ4:	What was the objective of the study?
EQ5:	What were the main results?
EQ6:	What was the sample size?
EQ7:	What was the ML validation method used?
EQ8:	What were the control group and psychiatric group?
EQ9:	What was the conclusion of the paper?
EQ10:	What was the limitation and future improvement?

### Quality assessment

The quality assessment of the current study was based on the reported evaluation metrics in the selected studies. In classification tasks, the most commonly used metrics to evaluate the performances of the models were accuracy (ratio of correct predictions to total number of predictions), precision (ratio of truth positive to total number of positive predictions), recall (ratio of truth positive to total number of actual positive cases; also known as sensitivity), F1-score (the harmonic mean of precision and recall), and Area Under the Receiver Operating Characteristics (the capability to distinguish different classes; also known as AUC-ROC or AUC). The choice of evaluation method depends on the task’s nature and the dataset’s distribution. In general, AUC is often used to compare performances between different models. Hence it is the primary measurement we will use to compare proposed models. For medical applications, where the cost of a false negative result can be extremely high, recall should be the main evaluation metric since a lower recall value implies a significant portion of ignored actual positive cases. For imbalanced datasets, where the number of each class varies significantly, the F1 score is essential since it considers both false positives and false negatives^21^. As for regression tasks, measures like mean absolute error (MAE), mean squared error (MSE), and root means squared error (RMSE) are significant^22^.

### Reporting summary

Further information on research design is available in the Nature Research Reporting Summary linked to this article.

## Results

### Study selection and review process

**Fig. 2:**
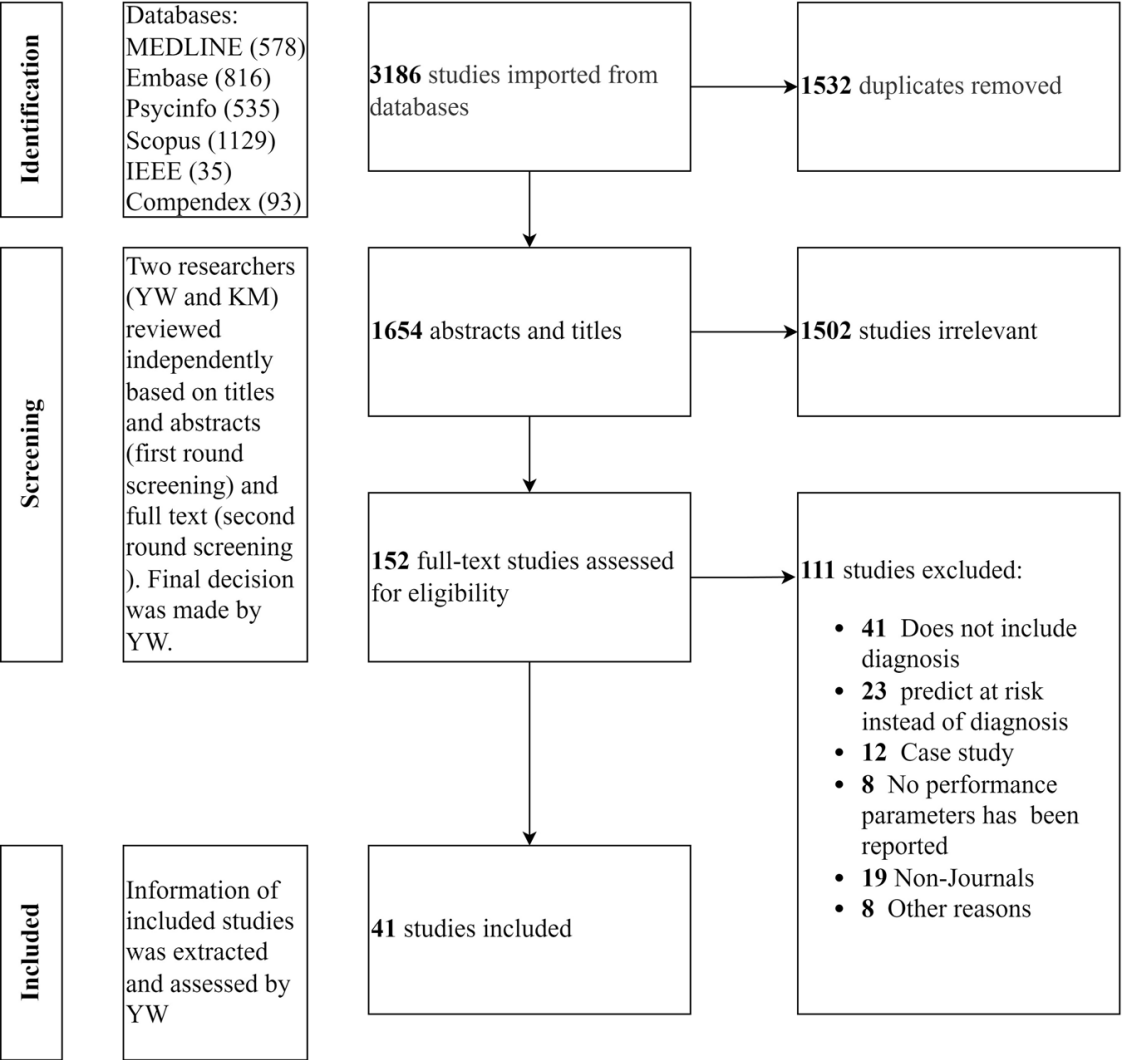
PRISMA flowchart of study selection and review process. This figure described the entire procedure of study selection based on the PRISMA systematic review guideline. We started with 3186 studies from six different databases. After duplication removal, abstract and title screening, and full-text screening, we finally ended up with 41 studies for this review. Detail selection process can refer to the Methods section.

The PRISMA guidelines were followed in our review of a total of 3186 studies (Fig. 2*PRISMA flowchart of study selection and review process*). The study screening process was efficiently managed by utilizing Covidence, an online systematic review management system. On import of these 3186 studies, Covidence seamlessly and automatically removed duplicate articles across various databases, yielding 1654 unique studies. Subsequently, a rigorous screening of titles and abstracts from these 1654 studies ensued, excluding 1502 studies that fell outside of the scope of this review. In a full-text screening of the remaining 152 studies, 111 studies were excluded, based on the inclusion and exclusion criteria outlined in Table 1. The final selection comprised 41 studies. The Supplementary Table 1 summarizes data extracted from these 41 studies, including 21 neuroimaging research studies^23–43^. Six of the studies used clinical interviews^44–49^, eight studies used data extracted from self-reported questionnaires or online surveys^50–57^, and six used blood markers, facial features, social media, GPS, or EMR to diagnose PTSD^58–63^. ML models were used in all studies to diagnose PTSD in diverse sample sets, including data collected from the general population as a heterogeneous group, patients who witnessed traumatic incidents, online databases, veterans, firefighters, and healthcare providers.

### Neuroimaging

A total of 21 included studies utilized neuroimaging techniques, including functional Magnetic Resonance Imaging (fMRI), Magnetoencephalography (MEG), and Electroencephalography (EEG), to perform automatic PTSD diagnosis across diverse PTSD sample groups.

Five studies conducted their experiments on heterogeneous sample groups. Nicholson et al. combined resting-state (rs) amplitude of low-frequency fluctuation (ALFF) and amygdala complex connectivity maps to detect PTSD via a multiclass Gaussian process classifier. Their model achieved a balanced accuracy of 96.08% for PTSD patients, indicating that increased amygdala activation was a strong indicator of PTSD^25^. Harricharan et al. also employed a multiclass Gaussian process classifier to identify PTSD cases. Their study used the anterior and posterior insula as predictive features, achieving an accuracy (ACC) above 0.80. They concluded that healthy controls (HCs) displayed enhanced connectivity between the insula and higher cortical brain regions related to environmental monitoring and emotion evaluation, particularly the left postcentral gyrus and left dorsolateral prefrontal cortex^26^. Zilcha-Mano et al. differentiated PTSD and major depressive disorder (MDD) patients from HCs using biomarkers from rest-state functional connectivity, achieving an accuracy of 70.6% and an AUC of 0.87 with an SVM classifier for PTSD participants. This study indicated that higher executive control network (ECN) connectivity was associated with more severe PTSD and MDD symptoms^29^. Nicholson et al. classified PTSD versus HCs during real-time fMRI neurofeedback (NFB) training with an accuracy of 80% and an AUC of 0.85 using the L1-Multiple Kernel Learning method. They concluded that PTSD and HCs groups exhibited decreased activity in the posterior cingulate cortex during neurofeedback (NFB) training and transfer runs^33^. Saba et al. compared the performance of various PTSD diagnostic ML models, discovering that the KNN method with train-dev-test accuracy rates of 96.6%, 94.8%, and 98.5%, respectively, and SVM with radial basis function kernel, with train-dev-test accuracy rates of 93.7%, 95.2%, and 99.2%, respectively, performed the best using predictive features from various brain regions^35^.

Four studies focused on diagnosing PTSD in individuals who had witnessed traumatic events. Gong et al. used an SVM classifier to differentiate between PTSD patients, HCs, and trauma-exposed non-PTSD groups (TE) based on gray and white matter predictors, which achieved an accuracy of 91% between PTSD and HCs and 67% between PTSD and TE, demonstrating that MRI data analysis can accurately differentiate PTSD participants from HCs^24^. Similarly, Zhang et al. employed an SVM classifier to distinguish PTSD from HCs, achieving an accuracy of 89.19% by using gray matter volume, ALFF, and regional homogeneity as predictors^27^. Using a DL approach, Yang et al. achieved a diagnostic accuracy of 71.2%, with a sensitivity of 0.60 and specificity of 0.83, by utilizing brain function groups such as frontoparietal areas, cingulate cortex, and amygdala as predictors^31^. Zhu et al. applied a graph-theoretic approach based on DL to discriminate PTSD from TE. An accuracy of 80% was achieved achieved with 0.81 sensitivity and 0.79 specificity by utilizing informative sets of brain graph measures of the central executive network, salience network, and default mode network^34^.

Four studies utilized ML to diagnose PTSD in veterans. Georgopoulos et al. used MEG to extract synchronous neural interactions (SNI) and differentiate PTSD from HCs using linear discriminant analysis (LDA) with bootstrap-based classification, achieving an accuracy of over 90%^23^. James et al. also employed SNI and an LDA classifier to identify PTSD in female veterans, reporting 100% accuracy and concluding that brain functional connectivity could be an objective indicator of recovery from PTSD^32^. Zhang et al. used MEG frequency bands (alpha, gamma) and an SVM classifier to classify male veteran PTSD, achieving an AUC of 0.90^28^. Shahzad et al. utilized the amygdala, hippocampus, and prefrontal cortex in the left and right brain hemispheres with an ANN to identify PTSD. The study found that the left hippocampus was the brain region most impacted by PTSD, and the right hippocampus was the least affected region. The study also reported an accuracy of 80.04% and an AUC of 0.88 with a specificity of 0.81 and sensitivity of 0.78 for the left brain, an accuracy of 93.03% and an AUC of 0.98 with a specificity of 0.97 and sensitivity of 0.89 for the right brain, and an accuracy of 94.12% and an AUC of 0.98 with a specificity of 0.91 and a sensitivity of 0.99 for both hemispheres^30^.

Eight studies, which adopted EEG as the diagnostic tool, predicted PTSD outcomes based on the electrical signals obtained from the device. Five studies had heterogeneous subject populations as their data resources. Shim et al. employed the P300 feature from EEG at both the sensor and source levels to differentiate between PTSD, MDD, and HCs using an SVM classifier. The study yielded an 80% accuracy, with 0.86 specificity and 0.72 sensitivity. The PTSD group exhibited significantly lower P300 amplitudes and longer latencies than the HCs group^36^. Kim et al. developed a novel ML classifier, the Fisher geodesic minimum distance to the mean (FgMDM), based on Riemannian geometry and combined it with EEG source covariance to distinguish between PTSD and HCs. The proposed model demonstrated an accuracy of 73.09% and 0.80 AUC, with 0.69 sensitivity and 0.77 specificity^37^. Park et al. used EEG parameters in 6 frequency bands to predict major psychiatric disorders, controlling for variables such as age, sex, education, and IQ background, achieving a predictive accuracy of 91.21% for PTSD^38^. By integrating microstate-based segmentation of various EEG frequencies with an SVM classifier, Terpou et al. integrated microstate-based segmentation of different EEG frequencies with an SVM classifier, distinguishing PTSD with an accuracy of 76%, AUC of 0.75, sensitivity of 0.79, and specificity of 0.74^39^. Shim et al.‘s 2022 study demonstrated that low-frequency EEG oscillations with an SVM classifier could increase the predictive accuracy to 86.61%, with an AUC of 0.93, analyzing resting-state EEG data at the source level in six frequency bands^40^.

Li et al. utilized EEG signals and features such as startle potentiation, fear generalization, fear extinction, and stimulus to predict PTSD in firefighters, achieving an AUC of 0.93 with sensitivity and specificity above 0.85 using a light gradient boosting classifier^41^. Breen et al. employed an SVM classifier to identify trauma-exposed PTSD by integrating features such as sleep disturbance, impaired declarative memory, and metabolite variables from polysomnogram recordings, achieving an accuracy of 80%^42^. Tahmasian et al. investigated subjective or objective sleep assessments as tools for automatic PTSD diagnosis. Using an SVM classifier, they performed a high classification accuracy of 91.6%, sensitivity of 0.93, and specificity of 0.90^43^.

Neuroimaging studies frequently use SVM (*n* = 9) for feature classification due to its ability to handle non-linear data, allowing high accuracy in models despite limited participant numbers. The correlation between neuroimaging features and patient psychiatric status simplifies feature engineering, reducing the need for complex methods like neural networks. Some studies demonstrated high accuracy using LDA combined with SNI, a robust biomarker for mental disorders such as PTSD.

fMRI scans focus on key brain regions, tissues, networks, and fluctuations like the global mean ALFF (mALFF). MEG scans study features from various bands and SNI, often outperforming resting-state fMRI. EEG measures the brain’s electrical signals correlating strongly with patients’ psychiatric states, making simpler ML models like SVM suitable. Predictive EEG features include frequency bands and their correlations, with several studies included in this review using quantitative EEG (QEEG) parameters, such as source-level Power Spectrum Density and Functional Connectivity, to classify PTSD from HCs.

### Structured clinical interviews

Six studies were conducted using interview data to automatically diagnose PTSD. He et al. utilized an ML product score model with lexical features to compare detected word scores between PTSD and HCs, achieving an 82% diagnostic accuracy with a sensitivity of 0.85 and specificity of 0.78^46^. Schultebrauck et al. used a DL-fused model to identify PTSD from individuals exposed to trauma, achieving a 0.90 AUC, 0.84 precision, 0.84 recall, and 0.83 F1 score by analyzing visual, acoustic, and semantic features from clinical interviews^47^. Two studies focused on using speech markers to diagnose PTSD in veteran populations. One of these (Marmar et al.), employed an RF classifier to analyze audio features both of which achieved an accuracy of 89.1%^45^. Three other clinical interviews examined in this review used speech and sentiment features from natural language to detect PTSD. Banerjee et al. utilized a deep belief network model (DBN) and transfer learning method on the TIMIT Speech Corpus to diagnose PTSD^64^ and achieved 74.99% accuracy, demonstrating its great potential for small datasets^44^. Gupata et al. tested an extreme gradient boosting (XGB) classifier on TIMIT and FEMH datasets for early PTSD diagnosis and achieved high accuracies of 97.5% and 96.29%, respectively^48^. Sawalha et al. utilized an RF classifier with a Vader semantic analyzer on semantic features from the Audio/Visual Emotion Challenge and Workshop (AVEC-19) corpus^65^ and reached 80.4% accuracy with 0.80 AUC^49^.

Speech features were the primary predictors in the clinical interview models, including acoustic features such as frequency and amplitude, prosodic features like rhythm, stress, and intonation, features related to the physical characteristics of the vocal tract, and excitation features associated with the vibration of the vocal cords. Transcripts from interview recordings were also utilized in some studies, and semantic features were extracted using textual analysis techniques such as bag-of-words. Unlike features extracted from neuroimaging, speech and textual features are more abstract and less specific for mental disorder diagnosis. Consequently, significant feature engineering is necessary to extract meaningful information from these features, making DL techniques suitable for classification. In fact, the clinical interviews considered here have shown a preference for DL models (*n* = 2) due to the sequential nature of speech and textual analysis. Researchers have used DL models combined with transfer learning to enhance classifiers’ performance significantly.

### Self-report questionnaires and narratives

Eight studies included in our review evaluated the effectiveness of self-report questionnaires and online surveys in detecting PTSD based on semantic features. He et al. used NLP and text mining to develop an automated PTSD screening tool, which incorporated a product score model and achieved an accuracy of 82% and an AUC of 0.94. Their findings demonstrated that self-narratives in text form could accurately assess PTSD, and the inclusion of higher-order n-grams improved classification metrics and prediction accuracy^57^.

Three of the self-report studies included patients with traumatic experiences. Kessler et al. utilized the ensemble ML model super learner to predict PTSD from data obtained from the World Health Organization’s world mental health survey, using features such as personal violence experiences and socio-demographics. They reported an AUC of 0.98^50^. Orovas et al. extracted data from self-report questionnaires, including demographics, prenatal and mental health variables, and used a multi-layer perception (MLP) classifier to analyze the data. They reported an accuracy of 92.9% for the PTSD group, with a precision of 0.83, recall of 0.89, and specificity of 0.98^52^. Bartal et al. examined whether a written narrative of childbirth experience from postpartum women could predict PTSD. They used an NLP model transformer and reported an AUC of 0.75, with an F1 score of 0.76, sensitivity of 0.8, and specificity of 0.7^56^.

Four self-report studies employed ML algorithms to detect PTSD in high-risk professionals such as veterans, firefighters, and healthcare workers. Karstoft et al. used an SVM classifier to identify pre- and post-deployment PTSD in a group of Danish soldiers, reporting an AUC of 0.84 and 0.88, respectively^53^. Portugal et al. developed a regression model to predict the severity of depression and PTSD in healthcare workers, using psychometric questionnaires and an SVM regressor with a mean square error of 0.90^51^. Campbell et al. utilized a decision tree (DT) classifier to predict unit-level risk for combat PTSD, achieving 90% accuracy by surveying veterans about their combat experiences^54^. Kim et al. predicted the prevalence of PTSD among firefighters using information such as suicide incidents and alcohol consumption with an SVM classifier. They obtained an accuracy of 89%, precision of 0.89, recall of 0.89, and F1 score of 0.89 when the support vector number was set to 20^55^.

Despite the abundance of self-reported questionnaire data, researchers often favored the SVM classifier over DL models. The most frequently used features in the self-reported questionnaire were demographics and data related to personal violence experiences, mental health conditions, PTSD symptoms, and traumatic experiences. SVM can effectively classify these well-engineered features obtained from surveys.

### Other diagnostic approaches

In addition to traditional interview-based and evidence-based diagnostic methods, this review includes six studies that used various innovative approaches as indicators for PTSD. One study performed automatic PTSD detection through social media. Ismail et al. applied CNN to PTSD diagnosis through keywords from Twitter and obtained an accuracy of 0.91 in the cancer survivor population^62^. With exon biomarkers, Tylee et al. achieved predictive accuracy of 90% when applying an SVM classifier^58^.

Two additional studies used RF classifiers to analyze medical records for automated PTSD diagnosis. Using similar predictors, Zafari et al. achieved an accuracy of 99%, specificity of 1.0, sensitivity of 0.78, F1 of 0.78, and AUC of 0.89. They concluded that using existing primary care data to detect PTSD can improve primary care quality, conduct research, and monitor patient health^61^. Gagnon-Sanschagrin et al. used an RF classifier with data on antiadrenergic medication use, bipolar disorder diagnosis, musculoskeletal and connective tissue diseases, substance use/abuse, and physiological symptoms or reactions to identify individuals with undiagnosed PTSD. An AUC of 0.75 was reported in this study^63^.

Gavrilescu et al. introduced a Facial Action Coding System, which extracts facial expression features from recordings to determine MDD, anxiety, and PTSD. They adopted an SVM classifier and obtained an accuracy of 90.2% for the PTSD group^59^. Lekkas et al. conducted an experiment on female trauma witnesses in which they used the daily time spent away from home and the maximum distance traveled from home to diagnose PTSD. By feeding the global positioning system (GPS) information into an XGB classifier, they achieved an AUC of 0.82, sensitivity of 0.74, specificity of 0.80, and accuracy of 77.1%^60^.

## Discussion

**Fig. 3:**
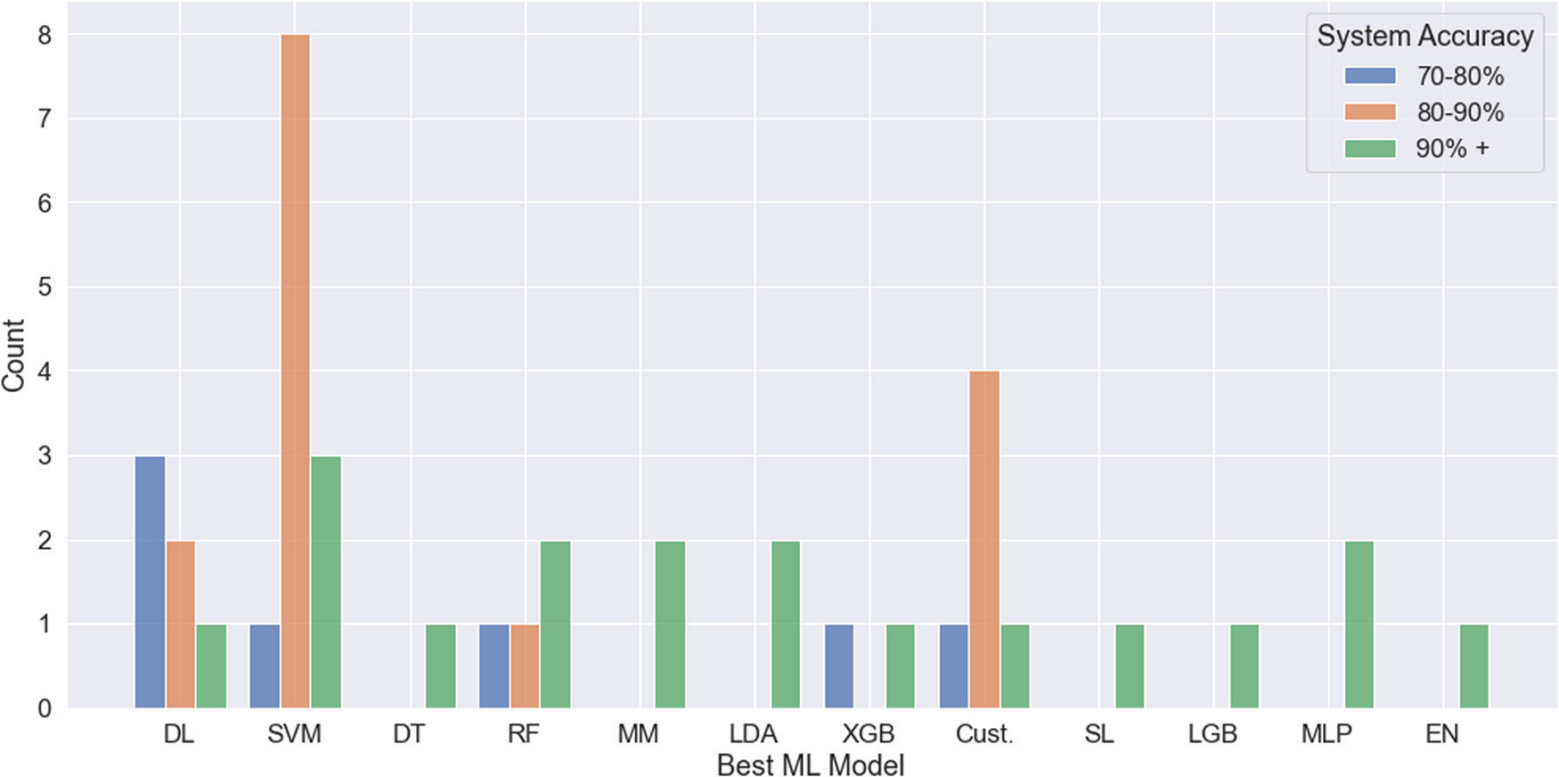
Histogram of various ML models with system accuracy. This figure counted the occurrence of each ML model the authors reported as the best model from the included studies. The blue bar represents model accuracy between 70–80%, the orange bar represents model accuracy between 80–90%, and the green bar represents model accuracy above 90%. Abbreviations: EN Elastic Net, SL SuperLearner, PCA Principal Component Analysis, Cust. Customized Model, LGB light GB, MM Multiple Models.

Our review analyzed 41 studies that utilized AI technologies for PTSD diagnosis. Among the ML models and validation methods used in these studies, SVM (*n* = 12) and k-fold cross-validation (*n* = 30) were the most commonly employed. Our statistical analysis indicates that SVM, DL, and combined models outperform others (Fig. 3*Histogram of various ML models with system accuracy*). SVM, a long-standing ML model, remains preferred for ML scientists due to its efficacy and suitability for small to moderate-sized datasets and its relatively low computational requirements. It is beneficial when the predictive features are well-known to the researchers. In recent years, DL models have grown increasingly popular with the expansion of data availability in various domains. The studies in our review utilized CNN and RNN models, such as Long Short-term memory (LSTM)^16^, as their classifiers, demonstrating the potential when sufficient sample sizes are available. DL models require less feature engineering than traditional ML models since the hidden layers can select informative features.

**Fig. 4:**
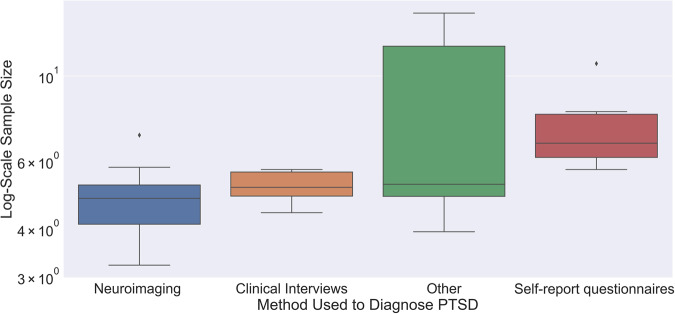
Sample distribution box plot of different PTSD diagnostic methods. This figure visualized the distribution of database sample size the authors adopted for ML training in log scale for different PTSD diagnostic tools. Outliers were indicated by the black solid diamond symbol. In each individual box, the upper horizontal line represented maximum sample size (excluding the outliers) while the bottom horizontal line represented minimum sample size (excluding the outliers). The “whiskers” are lines that extend from the box to the minimum and maximum values in the dataset. For the box, it represented the region where the majority sample size distribution (fist quartile to third quartile) with the central line representing median sample size. Different color represented different PTSD diagnostic method.

The sample size varied widely among the reviewed studies, ranging from 24 participants to 2,124,496 surveys, with a median of 179 samples. Figure 4 (*Sample distribution box plot of different PTSD diagnostic methods after removing extreme outliers*) displays the sample size distribution for each diagnostic method. Diagnostic methods like neuroimaging and clinical interviews tend to have limited sample sizes due to the high costs of using specialized equipment and trained professionals. Consequently, the sample sizes for these methods are often small, which can negatively impact the performance of the corresponding ML models. Despite this limitation, data collected through these methods is precise to PTSD as the predictive features strongly correlate with PTSD diagnosis. Therefore, these models are valuable in clinical settings where the likelihood of PTSD symptoms may be high.

**Fig. 5:**
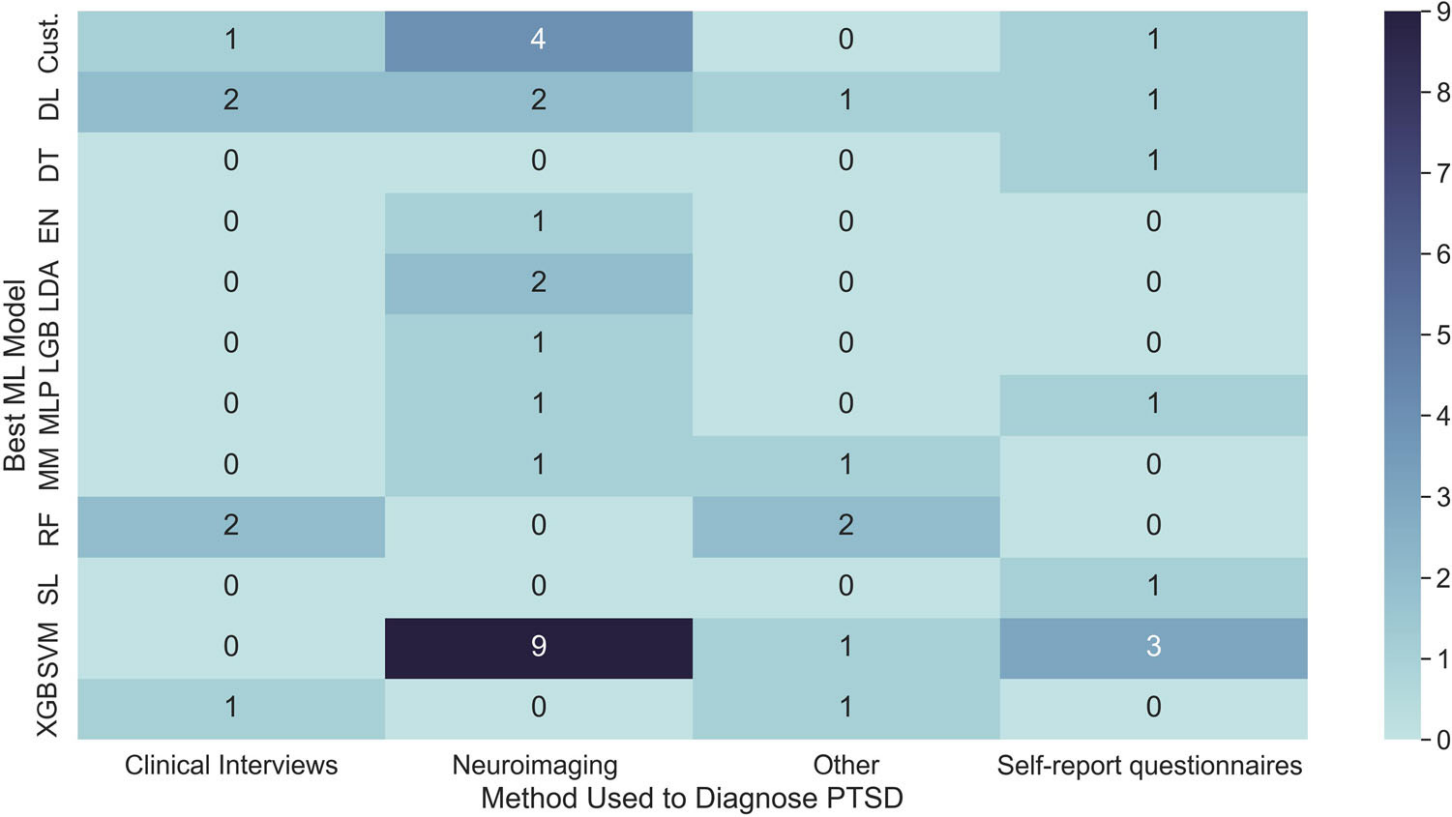
ML model counts in different PTSD diagnostic methods. This figure was the heatmap representation of ML model types vs. PTSD diagnostic methods. Each cell represented a ML model and diagnostic method pair with number indicating the occurrence in the included studies. In terms of color scales, darker blue indicated higher occurrence while brighter blue indicated lower occurrence.

In contrast to neuroimaging and clinical interviews, studies based on self-report questionnaires and other diagnostic methods generally have larger sample sizes. Many studies in these categories utilize online datasets or surveys, which are less expensive and allow for the accessible collection of large datasets. Other studies have used GPS information and electronic medical records, which provide access to extensive online databases containing significant amounts of data. More data available can often enhance the performance of the classification ability, particularly for DL models^66^. However, these data types are less specific to PTSD, and the samples are heterogeneous. Therefore, it can be challenging to extract useful predictive features specific to PTSD screening. The datasets are also at risk of data imbalance as PTSD-positive cases are often lower in number than HCs, resulting in overfitting of the ML model and losing the ability to generalize^67^. Figure 5 (*ML model counts in different PTSD diagnostic methods*) displays a heat map between ML model usage and PTSD diagnostic methods. Five factors identified from the literature impact the performance of ML models for PTSD classification: limited sample size (*n* = 13), comorbidity (*n* = 10), lack of generalizability (*n* = 7), insufficient study controls (*n* = 7), and imbalanced data distribution (*n* = 6). Overfitting due to limited sample sizes, particularly in neuroimaging and clinical interviews, decreases model accuracy, precision, and recall^68,69^. The impact of comorbidities like MDD and anxiety disorders on classifier performance is often not addressed^70^. Studies sometimes overlook medication use, diagnostic tools, or demographic backgrounds, introducing potential biases. Models lack generalizability when sample groups such as trauma witnesses or high-risk professionals do not represent the broader population. Data imbalance can lead to the under-representation of minority populations^71^.

Several challenges persist in AI applications for mental disorder diagnoses, including limited data availability and quality, the need for diversity in training data to avoid biased outcomes, and calls for increased AI model transparency and interpretability. Privacy and security considerations also hold paramount importance. Digital mental health applications must comply with ethical and legal guidelines, prioritizing data security and privacy, particularly for sensitive personal information like medical histories and psychological evaluations^72^. Unauthorized access, disclosure, and breaches must be prevented to maintain patient trust and promote healthcare engagement. Researchers are encouraged to share only extracted features and modify raw images or audio to remove identifiable information. Regulatory challenges and unresolved liability issues present another obstacle to adopting AI-powered mental disorder diagnoses. These considerations underscore the complex landscape that informs future PTSD classification research. With this review paper, we aim to guide future research in automated PTSD diagnosis, detailing ML model selection, predictive features, sample sizes, and validation methods. The choice of optimal ML models, primarily an empirical process, hinges on data suitability and the availability of computational resources.

Traditional ML models like SVM and ensemble models are recommended for data strongly correlated with PTSD diagnosis and requiring minimal feature engineering, such as neuroimaging data. These models are ideal for initial experimentation due to limited feature engineering requirements and lower computational demands. For intricate data like speech, visual, and textual features which necessitate more feature engineering, DL models or MLPs are often deemed appropriate. These models can parse complex features through hidden layers and activation functions, discerning correlations between the features and PTSD diagnosis automatically. Recently, DL models such as Transformer^17^, and its variant BERT^73^, have demonstrated outstanding performance in various NLP tasks, including audio and semantic analysis. These transformer-based models can understand the long-term dependencies in natural language samples via a self-attention mechanism, significantly improving the model’s ability to understand the context. Multi-modality ML models, proven effective in mental disorder-related tasks^74^, offer a holistic view by integrating visual, audio, and semantic modalities. This multi-view learning framework significantly enhances the model’s capabilities in mental health analysis.

Predictive features vary significantly across diagnostic methods. In neuroimaging, brain regions like the amygdala, hippocampus, prefrontal cortex, and insula provide robust predictive results when captured with fMRI techniques. Including SNI as a predictor is recommended when using MEG scans while exploring QEEG features such as PSD and FC are advised for EEG features. Various speech attributes like acoustic, prosodic, vocal tract, and excitation are important features in clinical interviews. Semantic elements are recommended for clinical interviews and self-report surveys, providing a comprehensive understanding of the individual and enhancing PTSD diagnosis accuracy.

In ML, data sample size and quality are paramount, particularly in medical applications where data acquisition can be complex and costly, often limiting the performance of ML models. Strategies to mitigate these effects include data augmentation techniques that synthesize new samples to enlarge the sample set, and transfer learning, which fine-tunes pre-trained models on smaller datasets. Crowdsourcing is another potential solution, facilitating cost-effective and time-efficient data collection and annotation.

Data imbalance is another challenge that can be addressed via several strategies. Resampling, either through oversampling the minority class or undersampling the majority class, can balance class distribution but at the risk of information loss^75^. Synthetic Minority Over-sampling Technique (SMOTE) is an alternative approach that generates synthetic data for the minority class by interpolating between existing instances^76^. Ensemble methods, including bagging and random forests, can alleviate data imbalance by aggregating predictions from multiple models.

Model validation is pivotal to ensure ML accuracy and reliability. Validation methods are problem-dependent, with binary classification problems frequently using metrics such as accuracy, precision, recall, and F1 score, and regression problems typically utilizing MSE and MAE. Alternative metrics such as AUC may be more suitable in skewed or imbalanced datasets. Particularly in medical applications, maximizing sensitivity to minimize false negatives is vital.

Cross-validation, a popular technique, reduces model variance by partitioning the data and training the model on some partitions while evaluating others. This provides a robust estimate of the model’s performance and offers insight into its generalization ability. K-fold cross-validation and leave-one-out cross-validation are popular methods, depending on the size of the available datasets and the computational resources. A permutation test is recommended to ensure model reliability and reduce stochastic effects^77^. Statistical significance can be determined by evaluating the model on the original and multiple permuted datasets, demonstrating that the model’s performance is not merely by chance.

This review has several limitations that need to be acknowledged. Firstly, the screening process only considered articles directly related to PTSD diagnosis, excluding those addressing prediction or identifying individuals at risk of PTSD. Therefore, further research is needed to understand the entire spectrum of PTSD diagnosis and prediction. Additionally, the limited number of articles (*n* = 41) included in this review restricts the ability to provide in-depth recommendations on PTSD diagnosis tasks. Moreover, the variability in sample groups, sample sizes, performance metrics used, and quality across the studies makes it challenging to make comprehensive comparisons and reach a conclusive outcome.

In conclusion, the heterogeneity of the method applied in each study and the lack of raw data limits our ability to conduct a meta-analysis for this review. A comprehensive synthesis and review of 41 studies concerning the automated diagnosis of PTSD using ML techniques were conducted in this study. With the increasing need for more cost-effective, reliable, and efficient methods for diagnosing PTSD, AI presents a promising solution to address this critical challenge, particularly for individuals who face difficulties accessing quality mental healthcare or experience stigma associated with seeking psychotherapy. The studies included in this review demonstrate the potential of AI for improving PTSD diagnostic approaches. To aid future efforts in automating PTSD classification, guidelines for model selection, feature selection, data acquisition, and validation methods are provided. However, despite advancements in meticulous feature engineering and model selection, the practical implementation of these systems still requires improvement. Significant barriers to the widespread clinical adoption and realization of the full potential of AI in early PTSD diagnosis include ethical and privacy considerations and the lack of standard regulations.

## Supplementary information

PRISMA Checklist

Reporting Summary

Supplementary Information

## Data Availability

Most of the data utilized in this study is included within the main body of this paper and the Supplementary Information. Any additional data that is not contained within these sections can be made available by the authors upon request.
