# Supplementary material for: Systematic review of machine learning in PTSD studies for automated diagnosis evaluation
Source: Npj Ment Health Res. 2023 Sep 27;2:16. doi: 10.1038/s44184-023-00035-w (PMC10955977; doi:10.1038/s44184-023-00035-w)
Supplement: Supplementary file 3 — Supplementary Information [file 44184_2023_35_MOESM3_ESM.pdf]

## Supplementary Information

## Supplementary Methods:

### Concepts and Keywords

1. PTSD:

    1. stress disorders, post-traumatic/

    2. (PTSD or PTSI or PTSS or ((posttraumatic or post traumatic or combat or trauma*) adj1 (growth or stress* or neurosis or neuroses or nightmare*)) or ((traumatic) adj (stress disorder* or stress symptom* or stress injur*)) or shell shock* or shellshock*).mp.

2. Artificial intelligence:

    (Artificial intelligence or AI or computer-assisted or image classification or image analysis or computer vision or deep learning or machine learning or natural language processing or NLP).mp.

    (Algorithm* AND computer*)?

3. Diagnosis/ Prediction

    sensitiv*.mp. OR specificity.mp. Or diagnos*.mp. OR di.fs. Or predict*.mp.

### DB1: Ovid MEDLINE(R) ALL <1946 to October 17, 2022>

1 stress disorders, post-traumatic/ 39597

2 (PTSD or PTSI or PTSS or ((posttraumatic or post traumatic or combat or trauma*) adj1 (growth or stress* or neurosis or neuroses or nightmare*)) or (traumatic adj (stress disorder* or stress symptom* or stress injur*)) or shell shock* or shellshock*).mp. 52069

3 1 or 2 61030

4 artificial intelligence/ or machine learning/ or deep learning/ or supervised machine learning/ or support vector machine/ or unsupervised machine learning/ or sentiment analysis/ 81411

5 (Artificial intelligence or AI or computer-assisted or image classification or image analysis or computer vision or deep learning or machine learning or natural language processing or NLP or Neural Network or speech recognition or pattern recognition or classification algorithm or support vector machine or random forest or classifier).mp. 647808

6 4 or 5 647851

7 (sensitiv* or specificity or diagnos*).mp. or di.fs. or predict*.mp. 8904558

8 3 and 6 and 7 578

### DB2: Embase <1974 to 2022 October 17> (OVID Interface)

1 exp posttraumatic stress disorder/ 74094

2 (PTSD or PTSI or PTSS or ((posttraumatic or post traumatic or combat or trauma*) adj1 (growth or stress* or neurosis or neuroses or nightmare*)) or (traumatic adj (stress disorder* or stress symptom* or stress injur*)) or shell shock* or shellshock*).mp. 87248

3 1 or 2 87365

4 machine learning/ or exp artificial neural network/ or automated pattern recognition/ or automatic speech recognition/ or exp back propagation/ or bayesian learning/ or exp classification algorithm/ or classifier/ or exp feature detection/ or feature extraction/ or exp "feature learning (machine learning)"/ or learning algorithm/ or network learning/ or random forest/ or semi supervised machine learning/ or supervised machine learning/ or exp support vector machine/ or unsupervised machine learning/ 224796

5 (Artificial intelligence or AI or computer-assisted or image classification or image analysis or computer vision or deep learning or machine learning or natural language processing or NLP or Neural Network or speech recognition or pattern recognition or classification algorithm or support vector machine or random forest or classifier).mp. 1525871

6 4 or 5 1537810

7 (sensitiv* or specificity or diagnos*).mp. or di.fs. or predict*.mp. 11020734

8 3 and 6 and 7 992

9 limit 8 to conference abstracts 176

10 8 not 9 816

## DB3: APA PsycInfo <1806 to October Week 2 2022>

1 posttraumatic stress disorder/ or "stress and trauma related disorders"/ or complex ptsd/ or desnos/ or posttraumatic stress/ or traumatic experiences/ 39992

2 (PTSD or PTSI or PTSS or ((posttraumatic or post traumatic or combat or trauma*) adj1 (growth or stress* or neurosis or neuroses or nightmare*)) or (traumatic adj (stress disorder* or stress symptom* or stress injur*)) or shell shock* or shellshock*).mp. 65069

3 1 or 2 65586

4 machine learning/ or artificial intelligence/ or exp machine learning algorithms/ or exp "pattern recognition (computer science)"/ or exp artificial neural networks/ or exp neural networks/ or exp text analysis/ 58297

5 (Artificial intelligence or AI or computer-assisted or image classification or image analysis or computer vision or deep learning or machine learning or natural language processing or NLP or Neural Network or speech recognition or pattern recognition or classification algorithm or support vector machine or random forest or classifier).mp. 128629

6 4 or 5 154635

7 (sensitiv* or specificity or diagnos* or predict*).mp. 1052949

8 3 and 6 and 7 535

## DB4: Scopus

Results: 1129

Date searched: Oct 18, 2022

TITLE-ABS-KEY(PTSD or PTSI or PTSS or ((posttraumatic or post-traumatic or combat or trauma*) W/1 (growth or stress* or neurosis or neuroses or nightmare*)) or (traumatic W/1 (stress-disorder* or stress-symptom* or stress-injur*)) or shell-shock* or shellshock*) AND TITLE-ABS-KEY(Artificial-intelligence or AI or computer-assisted or image-classification or image-analysis or computer-vision or deep-learning or machine-learning or natural-language-processing or NLP or Neural-Network or speech-recognition or pattern-recognition or classification-algorithm or support-vector-machine or random-forest or classifier) AND TITLE-ABS-KEY(sensitiv* or specificity or diagnos* or predict*)

## DB5: IEEE

Searched Oct 18, 2022

"Post traumatic stress disorder" and "machine learning" and diagnosis - 7 results

"Post traumatic stress disorder" and "machine learning" and predict* - 8 results

"Post traumatic stress disorder" and "deep learning" and predict* - 1 results

"Post traumatic stress disorder" and "deep learning" and diagnosis - 2 results

"Post traumatic stress disorder" and "artificial intelligence" and diagnosis - 7 results

"Post traumatic stress disorder" and "artificial intelligence" and predict* - 10 results

## DB6: Engineering Village (formerly compendex)

Searched Oct 18, 2022

Results: 93

SUBJECT/TITLE/ABSTRACT : (PTSD or traumatic-stress-disorder* or traumatic-stress-symptom* or traumatic-stress-injur* or shell-shock* or shellshock*)

AND

SUBJECT/TITLE/ABSTRACT : (Artificial-intelligence or AI or computer-assisted or image-classification or image-analysis or computer-vision or deep-learning or machine-learning or natural-language-processing or NLP or Neural-Network or speech-recognition or pattern-recognition or classification-algorithm or support-vector-machine or random-forest or classifier)

AND

SUBJECT/TITLE/ABSTRACT : (sensitiv* or specificity or diagnos* or predict*)

## Supplementary Table 1:

| | Diagnostic Method | Data Source | Best ML Model | Performance Metrices | Predictive Features | Sample Size | Validation Method |
|---|---|---|---|---|---|---|---|
| Banerjee et al. (44) | Clinical Interviews | TIMIT Speech | DL | ACC: 0.7499 | Acoustic features | 168 for speech | LOOCV |
| | | Corpus and | | | | and 26 for PTSD | |
| | | PTSD | | | | | |
| | | Speech Corpus | | | | diagnosis | |
| Marmar et al. (45) | Clinical Interviews | veteran | RF | ACC: 0.891 | Quality of speech | 129 samples (52 | cross-validation |
| | | | | AUC: 0.954 | | PTSD and | |
| | | | | | | 77 HC) | |
| He et al.(46) | Clinical Interviews | Heterogenous | product score | With threshold = 0: | Text mining results | 300 with 150 | 15 fold CV |
| | | | model | SEN: 0.85,SPE: 0.78 | Bag of words | PTSD and 150 | |
| | | | | ACC: 0.82 | | non-PTSD | |
| Schultebraucks et al. (47) | Clinical Interviews | trauma witness | DL | AUC: 0.90, PRE: 0.84 | video-based marker, | 81 patients | 10 fold CV |
| | | | | RECALL: 0.84 ,F1: 0.83 | audio-based marker, | | |
| | | | | | facial features | | |
| Gupata et al. (48) | Clinical Interviews | TIMIT and FEMH | XGB | TIMIT: ACC: 0.975, | Prosodic features, | not mentioned | not mentioned |
| | | dataset, | | SPE: 0.952, SEN: 0.945 | excitation features, | | |
| | | heterogenous | | RECALL: 0.938 | vocal tract features | | |
| | | | | FEHM: ACC: 0.9629, | | | |
| | | | | SPE:0.9353, SEN: 0.927 | | | |
| | | | | RECALL: 0.904 | | | |
| Sawalha et al. (49) | Clinical Interviews | AVEC-19 corpus, | RF combined | ACC: 0.804, AUC: 0.80 | Semantic features | 275 participants | 5 fold CV |

| | | | | | | | |
|---|---|---|---|---|---|---|---|
| | | Veterans (105 | Vader semantic | F1 for control: 0.85 | Binning different | (188 HC and 87 | |
| | | females) | analyzer | F1 for target: 0.72 | words, | PTSD) | |
| | | | outperforms the | | 23 bins are the best | | |
| | | | rest | | | | |
| Breen et al. (42) | Neuroimaging (EEG) | Trauma witness | SVM | ACC: 0.8 | subjective and | 60 participants | LOOCV |
| | | | | | objective | (20 PTSD, 20 TE | |
| | | | | | sleep, neutral | and 20 HC) | |
| | | | | | declarative memory | | |
| | | | | | , and metabolite | | |
| | | | | | variables. | | |
| Shim et al. (36) | Neuroimaging (EEG) | Heterogenous | SVM | ACC: 0.8 ,SEP: 0.8627 | P300 signal | 90 participants | LOOCV |
| | | | | SEN: 0.7179 | | (51 PTSD and 39 | |
| | | | | | | healthy) | |
| Kim et al. (37) | Neuroimaging (EEG) | Heterogenous | Riemannian | ACC: 0.7309 ,SEN: | EEG source | 81 participants | 10 fold CV |
| | | | geometry-based | 0.6872 SPE: 0.7714 | covariance | (39 HC and 42 | |
| | | | classifier | ,AUC:0.7970 | | PTSD) | |
| Park et al. (38) | Neuroimaging (EEG) | Heterogenous | Elastic net | ACC: 0.9121 | EEG parameters in | 223patients (95 | 10 fold CV |
| | | | | | each | healthy control | |
| | | | | | frequency band | and 128 PTSD) | |
| Terpou et al. (39) | Neuroimaging (EEG) | Heterogeneous | SVM | with alpha band | microstate-based | 122 participants | 10 fold CV |
| | | | | ACC:0.76 ,SEN:0.79 | segmentation of | (61 PTSD and 61 | |
| | | | | SPE:0.74 ,AUC:0.75 | various frequencies | HC) | |
| Shim et al. (40) | Neuroimaging (EEG) | Heterogenous | SVM | ACC: 0.8661 | PSDs were extracted | 135 participants | LOOCV |
| | | | | AUC: 0.93 | from 6 frequency bands | (77 PTSD and 58 | |

| | | | | | | | |
|---|---|---|---|---|---|---|---|
| | | | | | | HC) | |
| Li et al. (41) | Neuroimaging (EEG) | firefighters | Light GBM | AUC: 0.93 | EEG signals | 1107 participants | 5 fold CV |
| | | | model. Gradient | SEN and SPE >0.85 | startle potentiation feat, | | |
| | | | boosting | | generalization feat, | | |
| | | | decision tree | | fear extinction feat | | |
| | | | | | ,stimulus feat | | |
| Tahmasian et al. (43) | Neuroimaging (EEG) | veteran | SVM | ACC:0.916 | Subjective or | 64 participants | LOOCV |
| | | | | SEN:0.93, SPE:0.903 | objective | (32 PTSD, 32 | |
| | | | | | sleep assessment | HC) | |
| Georgopoulos et al. (23) | Neuroimaging | veteran | LDA | ACC >0.9 | SNI | 324 veterans | cross-validation |
| | (MEG) | | | | | | |
| Gong et al. (24) | Neuroimaging (MRI) | Disaster | SVM | PTSD vs HC: 91% | grey and white | 140 (50 PTSD, 50 | LOOCV |
| | | witness | | PTSD vs TE: 67% | matter | TE, 40 HC) | |
| Nicholson et al. (25) | Neuroimaging (MRI) | Heterogenous | Multiclass | Balanced ACC:0.9163 | resting-state mALFF, | 181 participants | LOOCV |
| | | | Gaussian Process | | amygdala complex | (81 PTSD, 49 | |
| | | | Classification | | connectivity maps | PTSD+DS, 51 HC) | |
| Harricharan et al. (26) | Neuroimaging (MRI) | Heterogenous | multiclass | ACC > 0.80 for HC, | anterior insula and | 184 participants | LOOCV |
| | | | Gaussian process | PTSD, | posterior insula | (84 PTSD, 49 | |
| | | | classification | and PTSD + DS | | PTSD+DS, 51 HC) | |
| Zhang et al. (27) | Neuroimaging (MRI) | Trauma witness | SVM | ACC: 0.8919 | GMV, ALFF, and | 57 participants: | LOOCV |
| | | | | AUC 0.90 | regional homogeneity | 17 PTSD, 20 TE | |

| | | | | | | | |
|---|---|---|---|---|---|---|---|
| | | | | | | and 20 HC | |
| Zhang et al. (28) | Neuroimaging | veteran (all male) | SVM | AUC: 0.90 | MEG frequency bands | 44 veterans (23 | 10 fold |
| | (MEG) | | | | (alpha, gamma...) | PTSD and 21 HC) | |
| Zilcha-Mano et al. (29) | Neuroimaging (MRI) | Heterogenous | SVM | ACC: 0.706 | ECN, SN | 179 participants | 10 fold CV |
| | | | | AUC 0.87 | | (51 PTSD, 52 | |
| | | | | | | PTSD+MD and | |
| | | | | | | 76TEHCs) | |
| Shahzad et al. (30) | Neuroimaging (MRI) | Veterans | ANN | Left brain : ACC: 0.8004, | the amygdala, | 28 individuals | 5 fold CV |
| | | | | AUC: 0.877, SPE: | hippocampus, and | with 14 PTSD and | |
| | | | | 0.8122 SEN: 0.7771 | medial prefrontal cortex | 14 HC | |
| | | | | Right brain : ACC: 0.9302, | in the left, right, and | | |
| | | | | AUC: 0.98, SPE: 0.9694 | both hemispheres. | | |
| | | | | SEN: 0.8933 | | | |
| | | | | Both brains : ACC: 0.9412, | | | |
| | | | | AUC: 0.984, SPE: 0.9103 | | | |
| | | | | SEN: 0.9942 | | | |
| Yang et al. (31) | Neuroimaging (MRI) | trauma witness | Deep learning | ACC:0.712 | Brain functioning groups | 86 participants | 10 fold CV |
| | | | | SEN: 0.597 | Frontoparietal areas | (33 PTSD, 53 HC) | |
| | | | | SPE: 0.827 | | | |
| James et al. (32) | Neuroimaging | Veteran | LDA | ACC: 100% | SNI | 121 veterans | LOOCV |
| | (MEG) | (all female) | | | | | |

| | | | | | | | |
|---|---|---|---|---|---|---|---|
| Nicholson et al. (33) | Neuroimaging (MRI) | Heterogenous | MKL | ACC: 0.8 | real-time fMRI | 29 patients (14 | LOOCV |
| | | | | AUC: 0.85 | neurofeedback, Brain | PTSD 15 HC) | |
| | | | | | regions | | |
| Zhu et al. (34) | Neuroimaging (MRI) | trauma witness | DL | ACC: 0.80 | Brain structures: CEN, | 217 participants | 5 fold CV |
| | | | | SEN: 0.809 | SN, DMN… | (91 PTSD and 126 | |
| | | | | SPE: 0.792 | | HC) | |
| Saba et al. (35) | Neuroimaging (MRI) | heterogeneous | KNN and SVM | KNN train-vali-test ACC: | brain regions: Prec TM | 24 patients ( 14 | validation set |
| | | | | (96.6%, 94.8%, 98.5%) | HC Amyg MPFC | PTSD 14 HC) | |
| | | | | SVM train-vali-test ACC: | | | |
| | | | | (93.7%, 95.2%, 99.2%) | | | |
| Tylee et al. (58) | Other: Blood | veterans | SVM | biomarker panel based on | peripheral blood | 50 patients (25 | 10 fold CV |
| | | | | 20 exons attained ACC:0.9 | biomarkers, exons | PTSD and 25 HC) | |
| Gavrilescu et al. (59) | Other: Facial Action | Heterogenous | AAM+SVM+FFNN | ACC: 0.902 | Facial expression | 128 Caucasians | not mentioned |
| | | | | | recording | with 17 PTSD | |
| Lekkas et al. (60) | Other: GPS | trama witness (all | XGB | AUC:0.816 | daily time spent away | 185 women | 10 fold CV |
| | | female) | | SEN:0.743 | and maximum | | |
| | | | | SPE:0.80 | distance traveled from | | |
| | | | | ACC:0.771 | home | | |
| Zafari et al. (61) | Other: Medical record or | Heterogenous | RF | ACC:0.99, SPE:1.0, | patient demographics, | 154118 patients | 10 fold CV |

| | | | | | | | |
|---|---|---|---|---|---|---|---|
| | personal record | | | SEN:0.78, F:0.78, | medical conditions, and | | |
| | | | | AUC:0.89 | financial conditions | | |
| Gagnon-Sanschagrin et al. (63) | Other: Medical record or personal record | IBM MarketScan Commercial Subset | RF | AUC: 0.75 | Personal medical records | 2124496 patients | not mentioned |
| Ismail et al. (62) | Other: social media | trauma witness | CNN | ACC: 0.9129 | Social media keywords | Not mentioned | 5 fold CV |
| Kessler et al. (50) | Self-report | Trauma witness | Superlearner | AUC: 0.96 | Personal experiences | 47466 surveys | 10 fold CV |
| | questionnaires | | | | | | |
| Karstoft et al. (53) | Self-report | veteran | SVM | Pre-deployment : | Personal conditions | 561 veterans | 10 fold CV |
| | questionnaires | | | AUC: 0.84 | | | |
| | | | | Post-deployment: | | | |
| | | | | AUC: 0.88 | | | |
| He et al. (57) | Self-report | Heterogeneous | product score | PSM: ACC: 0.82 , SPE: | n-gram. Verbal features, | 300 with 150 | 10 fold CV |
| | questionnaires | (mean age of 30.06 | model | 0.81 , AUC: 0.94 | text mining | PTSD and 150 non- | |
| | | ; 65%females) | | | | PTSD | |
| Portugal et al. (51) | Self-report | COVID-19 Health | SVM | Regression model | Predefined questions to | 437 participants | 5 fold and 2 fold |
| | questionnaires | workers(73.2% | | NMSE = 0.90 for 5 fold ; | access | | |
| | | female, mean age | | NMSE = 0.96 for 2 fold | | | |
| | | of 39.5 with SD of | | | | | |
| | | 10.8) | | | | | |

| | | | | | | | |
|---|---|---|---|---|---|---|---|
| Campbell et al. (54) | Self-report | veteran | DT | ACC: 0.9 | Combat Experiences | 3212 veterans | validation set |
| | questionnaires | | | | Scale items | (217 PTSD) | |
| Kim et al. (55) | Self-report | firefighter | SVM | ACC: 0.89, PER: 0.89 | ICE-R, CESD, Suicide | 2705 participants | 10 fold CV |
| | questionnaires | | | RECALL: 0.89, F1: 0.89 | Accident (SBQ-R), and | | |
| | | | | Support vectors: 20 | Alcohol Drinking Scale | | |
| | | | | | (AUDITK). | | |
| Orovas et al. (52) | Self-report | Trauma witness | MLP | For PTSD group: | Demographics, prenatal | 469 women | 10 fold CV |
| | questionnaires | | | PRE: 0.83, RECALL: 0.89 | health variables, mental | | |
| | | | | SPE: 0.98, ACC: 0.929 | health variables… | | |
| Bartal et al. (56) | Self-report | postpartum | DL(Transformer) | AUC: 0.75, F1: 0.76 | semantic features (NLP) | 1127 WOMEN (86 | 10 fold CV |
| | questionnaires | women | | SEN: 0.8, SPE: 0.7 | | PTSD) | |

Table 1: Selected information of included studies[1]

[1] Citation refers to the main manuscript. Abbreviations: EEG: Electroencephalogram; GPS: Global Positioning System; SVM: Support Vector Machine; GBM: Gradient Boosting Model; NB: Naive Bayes; DL: Deep Learning; RF: Random Forest; DT: Decision Tree; MLP: Multilayer Perceptron; XGB: Extreme Gradient Boosting; CNN: Convolutional Neural Network; LSTM: Long Short-term Memory; LDA: Linear Discriminate Analysis; KNN: K-nearest Neighbours; PCA: Principal Component Analysis; AAN: Active Appearance Models; FFNN: Feedforward Neural Network; SMO: Sequential Minimal Optimization; GRU: Gated Recurrent Unit; EE: Easy Ensembles; ACC: Accuracy; AUC: Area Under ROC Curve; SPE: Specificity; SEN: Sensitivity; PRE: Precision; PLV: Phase Locking Values; QEEG: Quantitative EEG; PSDs: Power Spectrum Densities; FC: Functional Connectivities; PSG: Polysomnogram MFCC: Mel-frequency Cepstral Coefficients; ALFF: Amplitude of Low-frequency Fluctuations; GMV: Gray Matter Volume; GM: Gray Matter; WM: White Matter; MEG: Magnetoencephalography; ECN: Executive Control Network; SN: Salience Network; DMN: Default Mode Network. LIWC: Linguistic Inquiry and Word Count; POF: Pattern of life; NLP: Natrual Language Processing; MD: Major Depression; HC: Healthy Controls; TE/TEC: Trauma Exposed Controls; LOOCV: Leave-one-out Cross-validation.
